# Single-Unit Muscle Sympathetic Nerve Activity Reflects Sleep Apnea Severity, Especially in Severe Obstructive Sleep Apnea Patients

**DOI:** 10.3389/fphys.2016.00066

**Published:** 2016-03-02

**Authors:** Takuto Hamaoka, Hisayoshi Murai, Shuichi Kaneko, Soichiro Usui, Yoshitaka Okabe, Hideki Tokuhisa, Takeshi Kato, Hiroshi Furusho, Yu Sugiyama, Yasuto Nakatsumi, Shigeo Takata, Masayuki Takamura

**Affiliations:** ^1^Department of Disease Control and Homeostasis, Graduate School of Medical Science, Kanazawa UniversityKanazawa, Japan; ^2^Kanazawa Municipal HospitalKanazawa, Japan

**Keywords:** microneurography, sleep apnea syndromes, muscle sympathetic nerve activity, single-unit recordings, apnea-hypopnea index

## Abstract

Obstructive sleep apnea syndrome (OSAS) is associated with augmented sympathetic nerve activity, as assessed by multi-unit muscle sympathetic nerve activity (MSNA). However, it is still unclear whether single-unit MSNA is a better reflection of sleep apnea severity according to the apnea-hypopnea index (AHI). One hundred and two OSAS patients underwent full polysomnography and single- and multi-unit MSNA measurements. Univariate and multivariate regression analysis were performed to determine which parameters correlated with OSAS severity, which was defined by the AHI. Single- and multi-unit MSNA were significantly and positively correlated with AHI severity. The AHI was also significantly correlated with multi-unit MSNA burst frequency (*r* = 0.437, *p* < 0.0001) and single-unit MSNA spike frequency (*r* = 0.632, *p* < 0.0001). Multivariable analysis revealed that SF was correlated most significantly with AHI (*T* = 7.27, *p* < 0.0001). The distributions of multiple single-unit spikes per one cardiac interval did not differ between patients with an AHI of <30 and those with and AHI of 30–55 events/h; however, the pattern of each multiple spike firing were significantly higher in patients with an AHI of >55. These results suggest that sympathetic nerve activity is associated with sleep apnea severity. In addition, single-unit MSNA is a more accurate reflection of sleep apnea severity with alternation of the firing pattern, especially in patients with very severe OSAS.

## Introduction

Obstructive sleep apnea syndrome (OSAS) is a known risk factor for cardiovascular events (Mooe et al., 1996), and increased sympathetic nerve activity can worsen the mortality and severity of both cardiovascular disease and OSAS. Several mechanisms are hypothesized to increase sympathetic nerve activity in OSAS patients. In patients with OSAS, blood oxygenation levels decrease during sleep, and carbon dioxide levels increase as a result, and these reactions stimulate peripheral chemoreceptors to increase central sympathetic nerve activity (Leuenberger et al., 1995; Morgan et al., 1995). In addition, afferent vagal signals from pulmonary stretch receptors are diminished, resulting in augmented sympathetic nerve activity (Bradley et al., 2003). The decrease in blood pressure and oxygenation also shift the set point of the arterial baroreceptors to the high pressure range, and as a result, blood pressure is increased both during the day and night (Cooper et al., 2005, 2007). These results suggest that decreasing sympathetic nerve activity and improving hypoxia are both important when treating OSAS.

To assess sympathetic nerve activity, muscle sympathetic nerve activity (MSNA), a direct recording of efferent sympathetic nerve activity, is still recognized as the gold standard method (Vallbo et al., 1979). Multi-unit MSNA frequency (BF) and incidence (BI) are generally used to assess sympathetic nerve activity quantitatively; however, the multi-unit MSNA index may underestimate the actual degree of central sympathetic nerve signaling to peripheral organs. Recently, we demonstrated that single-unit MSNA is more accurate than multi-unit MSNA, especially in intense sympathoexcitation states such as heart failure (Murai et al., 2006, 2009; Ikeda et al., 2012). Elam et al. (2002) reported that the number of multiple firings during one cardiac interval in OSAS patients was significantly increased compared with systolic heart failure (HF) patients, although multi-unit MSNA levels were similar between groups. Despite the interesting results from this study, only eight OSAS patients were included, so interpreting the relationship between sleep parameters and single-unit MSNA may be hard.

The apnea-hypopnea index (AHI), which is used to grade the severity of apnea in OSAS patients, is strongly related to mortality risk (Young et al., 2008). To date, little research has been performed using multi- and single-unit MSNA to examine the relationship between AHI and sympathetic nerve activity in OSAS patients. In addition, the degree to which the firing characteristics of central sympathetic nerve activity to the periphery is associated with AHI severity is unclear. In this study, we hypothesized that single-unit MSNA assessment in OSAS patients would provide more accurate information regarding disease severity compared with multi-unit MSNA.

## Methods

### Subjects

OSAS was diagnosed in patients with an AHI of ≥15 or ≥5 with daytime sleepiness. Patients who had more than five central sleep apnea events per hour were excluded. Patients were also excluded if they had HF, unstable angina pectoris, myocardial infarction, and/or coronary revascularization within 4 weeks of the study. HF was defined in accordance with the American Heart Association/American College of Cardiology guidelines (Hunt et al., 2009). Briefly, HF was diagnosed if patients had a left ventricular ejection fraction (LVEF) of ≤40% despite optimal treatment for at least 1 month or an LVEF of >40% with a history of acute pulmonary edema after excluding other non-cardiogenic diseases. Patients with atrial fibrillation or any implanted pacemaker devices, including implantable cardioverter defibrillators or cardiac resynchronization therapy, were excluded. Patients with an estimated glomerular filtration rate of <30 ml/min/1.73 m^2^ were also excluded. These conditions can affect sympathetic nerve activity (Hausberg et al., 2002; Hogarth et al., 2006).

This protocol was approved by the Research Ethics Board of the University of Kanazawa (Kanazawa, Japan). This study has been registered in the University Hospital Medical Information Network Center (UMIN, TOKYO, Japan) Clinical Trials Registration System as UMIN000017612. All patients provided informed written consent.

### Experimental protocols

This study was designed as a cross-sectional, observational trial. All investigators that assessed MSNA and PSG data were blinded to each patient's severity and characteristics.

### Polysomnography

PSG monitoring was performed overnight in the Sleep Disorders Laboratory of Kanazawa municipal hospital using an Embla N7000 system (Natus, San Carlos, CA, USA). Electroencephalogram (R&K method), right and left electrooculogram, body position, thoracic, and abdominal wall motion (Respiratory Inductive Plethysmogram system), electrocardiogram, or nasal airflow (Thermistor and pressure sensor), and oxygen saturation, measured using a finger probe pulse oximeter (Nonin 8000J Adult Flex Sensor, Plymouth, MN, USA), and the patient's sleep state were recorded during the session. Experienced investigators subsequently analyzed these data using Rembrandt analysis software (Natus, San Carlos, CA, USA). This inspection method was included in the type 1 category of the American Academy of Sleep Medicine, American Thoracic Society and the American College of Chest Physicians manuals for the examination of suspected sleep apnea in adults (Chesson et al., 2003). Patients were admitted to the laboratory at 16:00 on the day of measurement, and preparation for PSG started at 20:00. Measurement started at 21:00 after the lights were turned off and continued till 6:00 the next morning. All polysomnography measurements were supervised by a physician who was registered as a polysomnographic technologist by the American Academy of Sleep Medicine and a physician certified in sleep medicine by the Japanese Society of Sleep Research. Technicians were also qualified and certified.

### Apnea hypopnea index

The AHI was determined using standard criteria, which are described briefly below (Berry et al., 2012). An apnea event was scored when both of the following conditions were met: (1) a ≥90% drop in the peak signal, measured by an oronasal thermal sensor, compared with pre-event baseline levels (2) that was ≥10 s in duration. Apnea events with a long duration or increased inspiratory effort were defined as obstructive apnea events, and lack of inspiratory effort during the apnea event was defined as a central apnea event. If inspiratory effort was diminished and resumed later in the apnea event, it was scored as a mixed apnea event. A hypopnea event was scored if all of the following were met: (1) the peak signal excursions dropped by ≥30% of pre-event baseline using nasal pressure, (2) the duration of the ≥30% drop in signal excursion was ≥10 s, and (3) there was ≥3% oxygen desaturation from pre-event baseline or the event was associated with an arousal. Obstructive hypopnea was defined when at least one of the following was met during a hypopnea event: (1) snoring, (2) flattening of the nasal pressure, or (3) paradoxical motion of the chest and abdominal band excursions on respiratory inductance plethysmography. Hypopnea events that lacked these characteristics were defined as central hypopnea events. The AHI was defined as the number of episodes of apnea and hypopnea per hour of sleep.

### Muscle sympathetic nerve activity

MSNA recordings were taken in patients who had been diagnosed with OSAS within 1 week after polysomnography. All data were collected in the morning at 10:00. All participants were asked to abstain from alcohol and caffeine for 24 h and were tested at least 12 h post-prandial. Postganglionic MSNA was recorded from the right peroneal nerve at the fibular head using a high-impedance (10 MΩ) tungsten microelectrode. As described previously (Murai et al., 2006, 2009; Ikeda et al., 2012; Millar et al., 2015), the common peroneal nerve was detected by palpation and stimulated electrically at the skin surface. Investigators inserted a tungsten microelectrode percutaneously into a motor fascicle of the peroneal nerve. The microelectrode was adjusted until spontaneous pulse-synchronous, multi-unit bursts of sympathetic nervous activity could be validated. The microelectrode was adjusted further until a large unitary spike could be distinguished from background noise in the recording, which allowed for single-unit MSNA analysis. Single- and multi-unit MSNAs were recorded simultaneously from the same microelectrode. After a 15-min stabilization period, data were acquired over at least 5 min.

The electrodes were connected to a preamplifier at a gain of 1000 and to an amplifier at a gain of 70. The signal was fed through a band-pass filter (500–3000 Hz) and a resistance-capacitance integrated circuit with a time constant of 0.1 s to produce a mean voltage neurogram using a Power Lab recoding system (Model ML 785/85P; ADI Instruments, Bella Vista, Australia). The raw nerve signal was obtained at 12 kHz; other signals were obtained at 1000 Hz.

Once offline, experienced investigators identified multi-unit MSNA peaks in the integrated nerve recording based on the relationship with cardiac activity in a blinded fashion. Multi-unit MSNA was expressed as the number of bursts per minute (burst frequency: BF) and the number of bursts per 100 heartbeats (burst incidence: BI).

**Figure 1:**
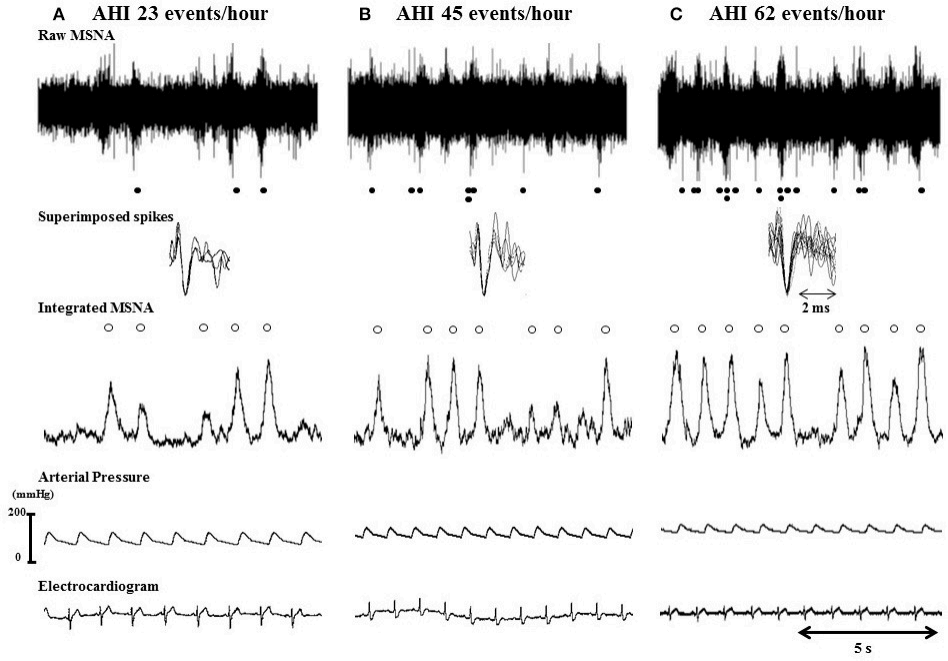
**Typical recordings of single- and multi-unit MSNA in three OSAS patients separated by OSAS severity (AHI). (A–C)** Show traces of single- and multi-unit MSNA in an OSAS patient with an AHI of 5–30 events/h, a severe OSAS patient (AHI of 30–55 events/h), and a very severe OSAS patient (AHI of >55 events/h), respectively. White circles indicate multi-unit MSNAs, and black circles indicate single-unit MSNAs. Single-unit MSNAs were confirmed by superimposing the action potentials.

Figure 1 shows typical single- and multi-unit integrated nerve recordings from OSAS patients divided by OSAS severity (AHI). When the raw neurogram record was clear enough to identify single-unit MSNA spikes, spike morphology was carefully inspected by experienced investigators. Single-unit spikes were defined as (1) spike synchronization with multi-unit MSNA bursts, (2) triphasic spike morphology with a negative main phase, and (3) superimposition of candidate action potentials with minimal variation. Single-unit MSNA was expressed as the number per minute (spike firing frequency: SF) and the number per 100 heartbeats (spike firing incidence: SI). Firing probability was defined as the percentage of heartbeats during which one or more spikes occurred. The firing probability was calculated from the number of cardiac intervals showing single-unit spikes divided by all cardiac intervals. The probability of multiple spikes was calculated from the number of cardiac intervals in which multiple spikes (two, three, or four single-unit spikes) were fired divided by all cardiac intervals with at least one spike. In addition, the percentage of cardiac intervals showing one, two, three, or four single-unit spikes was calculated from the number of cardiac intervals in which each single-unit spike were fired divided by all cardiac intervals with at least one spike. In this study, four was the maximum number of single-unit MSNA spikes during one cardiac cycle, which was the same as our previous study and the same as others have reported (Elam et al., 2002; Murai et al., 2012). The inter- and intra-observer correlations of multi-unit MSNA in this study were 0.890 and 0.91, respectively (*p* < 0.001) and for single-unit MSNA were 0.88 and 0.91 (*p* < 0.001). The reproducibility of single-unit MSNA has also been reported in previous reports (Lambert et al., 2011, Hering et al., 2013).

### Statistical analysis

All data are presented as means ± SD. Statistical analysis was performed using SPSS for Windows (version 17.0; SPSS Japan Inc. Tokyo, Japan) and EZR (Saitama Medical Center, Jichi Medical University, Saitama, Japan), which is a graphical user interface for R (The R Foundation for Statistical Computing, Vienna, Austria). More precisely, it is a modified version of R commander designed to add statistical functions frequently used in biostatistics. Univariate regression analysis was performed to detect correlations between the AHI and other clinical variables including MSNAs. Based on the results of the univariate analysis, a multiple regression analysis with stepwise backward selection was performed to determine the contribution of clinical variables to AHI severity. A *p*-value of ≥0.05 was an exclusion criteria for the stepwise procedure. In univariate and multivariate analysis, dummy variables are used in the analysis of nominal variables to include nominal variables into multivariate regression analysis. One-way ANOVA with Tukey's *post-hoc* test was used to analyze differences between groups. Pearson correlation coefficients were used to assess associations between study parameters. For all analyses, *p* < 0.05 (two-sided) was considered statistically significant.

## Results

PSG measurements were performed for the first time in 186 OSAS patients at our sleep disorder lab (Sleep Disorders Laboratory of Kanazawa Municipal Hospital) from June 2012 to April 2014. Of these patients, multi-unit MSNA was performed in 148. Data from 46 patients were excluded because a single-unit MSNA reading could not be detected; this was due to a low signal-to-noise ratio. One hundred and two OSAS patients were finally included in this study. Characteristics of the study population are shown in Table 1. The study population consisted of 75% (*n* = 77) males and 25% (*n* = 25) females, with a mean age of 57 ± 13 years and mean body mass index (BMI) of 25.9 ± 4.22 kg/m^2^. The prevalence of hypertension was 57.8% (*n* = 59); 8.82% (*n* = 9) of patients had diabetes, and 29.4% (*n* = 30) had dyslipidemia. The mean AHI was 34.5 ± 21.4 events/h, mean BF was 54.8 ± 12.2 bursts/min, mean BI was 81.1 ± 13.0 bursts/100 heartbeats, mean SF was 57.7 ± 12.9 spikes/min, and mean SI was 85.0 ± 17.1 spikes/100 heartbeats.

**Table 1:** **Baseline characteristics**.

**CLINICAL CHARACTERISTICS**	
Age (years)	57 ± 13
Female/Male	25/77
BMI (kg/m2)	25.9 ± 4.22
Hypertension (n, %)	59 (57.8)
Diabetes mellitus (n, %)	9 (8.82)
Dyslipidemia (n, %)	30 (29.4)
Systolic blood pressure (mmHg)	132 ± 16.5
Diastolic blood pressure (mmHg)	81.3 ± 12.2
BF (bursts/min)	54.8 ± 12.2
BI (bursts/100 heart beats)	81.1 ± 13.0
SF (spikes/min)	57.7 ± 12.9
SI (spikes/100 heart beats)	85.0 ± 17.1
**SLEEP PARAMETERS**	
AHI (events/h)	34.5 ± 21.4
ESS	8.14 ± 4.98
SREM (%)	17.3 ± 6.79
SES (%)	73.8 ± 22.9
Arousal index (events/h)	38.3 ± 20.7
3%ODI (events/h)	29.3 ± 22.7
Minimum SpO2 (%)	80.1 ± 8.03
Slow wave sleep (%)	3.58 ± 7.02
**MEDICATION (n, %)**	
ARB or ACEI	33 (32.4)
Calcium antagonist	30 (29.4)
β blocker	7 (6.86)
Diuretic	5 (4.90)
Statin	23 (22.5)

*Values are means ± SD. BMI, Body mass index; BF, burst frequency; BI, burst incidence; SF, spike firing frequency; SI, spike firing incidence; AHI, apnea hypopnea index; ESS, Epworth sleepiness scale; SREM, Stage REM; SES, sleep efficiency score; ODI, O_2_ desaturation index; ARB, angiotensin II receptor blocker; ACEI, angiotensin converting enzyme inhibitor*.

### Relationship between AHI and single- and multi-unit MSNA

**Figure 2:**
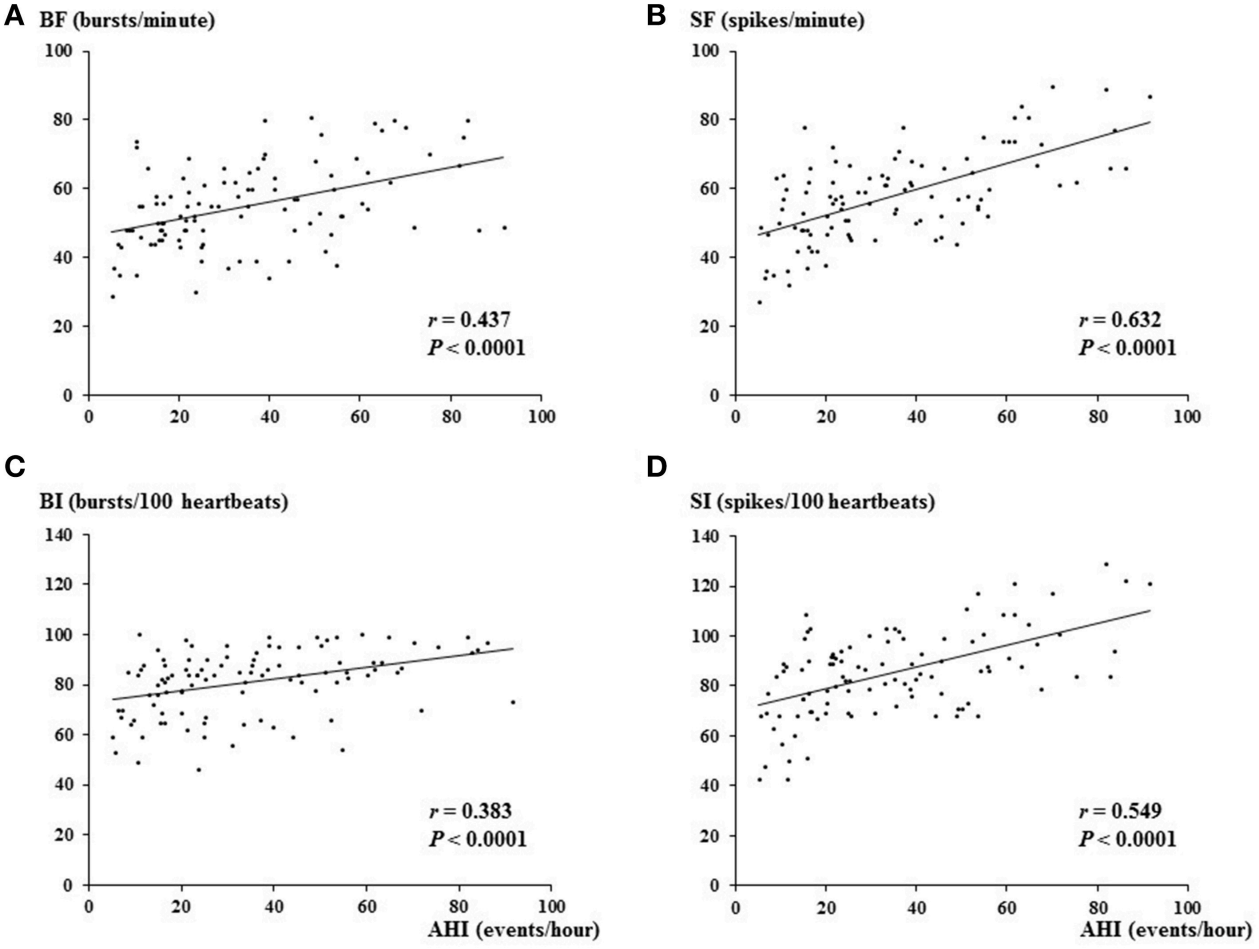
**Relationship between AHI and multi-unit MSNA (A, burst frequency; C, burst incidence) or single-unit MSNA (B, spike frequency; D, spike incidence)**. The correlation coefficient between single-unit MSNA and AHI was stronger than that between multi-unit MSNA and AHI. AHI, apnea-hypopnea index; BF, burst frequency; SF, spike frequency; BI, burst incidence; SI, spike incidence.

The correlation coefficients between AHI and each clinical parameter that may be affected by AHI tested by univariate and multivariate regression analyses are shown in Table 2. As expected, univariate analysis showed that multi-unit MSNA, expressed as BF and BI, was strongly correlated with AHI (*p* < 0.001; Figure 2). Single-unit MSNA frequency and incidence were also significantly correlated with AHI. Interestingly, the coefficients were higher between AHI and single-unit MSNA than between AHI and multi-unit MSNA. In addition, BMI and systolic and diastolic blood pressure were correlated with AHI. However, as shown in Table 2, multivariate analysis with stepwise method revealed that SF, BMI, and diastolic blood pressure were associated with AHI; however, SF had the most strongly significant relationship with AHI (*T* = 7.27, *p* < 0.0001).

**Table 2:** **Regression analysis between AHI and clinical parameters**.

**AHI**						
**Univariate regression analysis**			**Multivariate regression analysis**			
	*R*	*P*	β	*T*	*P*	VIF
BF	0.437	<0.0001				
SF	0.632	<0.0001	0.479	7.27	<0.0001	1.13
BMI	0.580	<0.0001	0.360	5.37	<0.0001	1.17
DM	0.192	0.052				
DL	0.128	0.200				
Gender	0.217	0.029				
Age	0.084	0.399				
SBP	0.370	<0.0001				
DBP	0.408	<0.0001	0.276	4.35	<0.0001	1.05

*AHI, apnea hypopnea index; BF, burst frequency (bursts/ min); BMI, Body mass index (kg/m^2^); DM, diabetes mellitus (with or without); DL, dyslipidemia (with or without); SBP, systolic blood pressure (mmHg); DBP, diastolic blood pressure (mmHg); VIF, variance inflation factor. β-values show standardized regression coefficients. T-values mean t-statistic*.

### Comparison of baseline characteristics according to AHI

Patients were divided into three groups according to AHI. The least severe group (S) comprised patients with an AHI of 5–30 events/h, the severe OSAS (SS) group comprised patients with an AHI of 30–55 events/h, and the very severe OSAS (VSS) group comprised patients with >55 events/h. Patient characteristics for each group are shown in Table 3. There was no significant difference in age among the three groups. The VSS and SS groups had more males than the S group. The VSS group had the highest BMI, which was significantly different from those of both the SS and S groups. As predicted, sleep parameters were significantly worse in both the VSS and SS groups than in the S group. In addition, systolic and diastolic blood pressure were significantly higher in the VSS and SS groups than in the S group. The proportion of patients treated with antihypertensive drugs and/or statins were similar among the three groups.

**Table 3:** **Comparison of baseline characteristics according to OSAS severity**.

**Events/h**	**S: 5~30 (*n* = 52)**	**SS: 30~55 (*n* = 32)**	**VSS: 55~ (*n* = 18)**
**CLINICAL CHARACTERISTICS**			
Age	57 ± 14	61 ± 10	52 ± 13
Male (n, %)	18 (34.6)	27 (84.4)^*^	15 (83.3)^*^
BMI (kg/m2)	23.9 ± 3.17	27.0 ± 4.01	29.5 ± 4.27^*^^#^
Hypertension (n, %)	28 (53.8)	20 (62.5)	11 (61.1)
Diabetes mellitus (n, %)	1 (1.92)	5 (15.6)^*^	3 (16.7)
Dyslipidemia (n, %)	17 (32.7)	10 (31.3)	3 (16.7)
Systolic blood pressure (mmHg)	126 ± 15.4	135.3 ± 14.1^*^	142.5 ± 17.9^*^
Diastolic blood pressure (mmHg)	77.3 ± 10.6	84.3 ± 10.6^*^	87.6 ± 15.2^*^
**SLEEP PARAMETERS**			
AHI (events/h)	17.4 ± 6.67	42.5 ± 7.51^*^	70.0 ± 11.1^*^^#^
ESS	8.10 ± 5.05	7.31 ± 4.75	9.72 ± 5.06^*^
SREM (%)	18.2 ± 7.45	16.7 ± 4.60	15.6 ± 7.94
SES (%)	78.0 ± 26.3	70.9 ± 16.6^*^	66.8 ± 20.2^*^
Arousal index (events/h)	23.8 ± 8.96	43.0 ± 11.2^*^	71.9 ± 13.9^*^^#^
3% ODI (events/h)	12.2 ± 6.93	36.3 ± 11.2^*^	66.5 ± 15.5^*^^#^
Minimum SpO2 (%)	83.9 ± 5.29	78.6 ± 6.92^*^	71.6 ± 9.19^*^^#^
**MEDICATION (n, %)**			
ARB or ACEI	19 (36.5)	9 (28.1)	5 (27.8)
Calcium antagonist	14 (26.9)	12 (37.5)	4 (22.2)
β blocker	3 (5.77)	3 (9.38)	1 (5.56)
Diuretic	1 (1.92)	3 (9.38)	1 (5.56)
Statin	15 (28.8)	4 (12.5)	4 (22.2)

*Values are means ± SD. BMI, Body mass index; BF, burst frequency; BI, burst incidence; SF, spike firing frequency; SI, spike firing incidence; AHI, apnea hypopnea index; ESS, Epworth sleepiness scale; SREM, Stage REM; SES, sleep efficiency score; ODI, O_2_ desaturation index; ARB, angiotensin II receptor blocker; ACEI, angiotensin converting enzyme inhibitor*.

* *p < 0.05, compared to S group*;

# *p < 0.05, compared to SS group*.

### Differences in single- and multi-unit MSNA among the three groups

Both single- and multi-unit MSNA were significantly augmented in the VSS group compared with the SS and S groups, as shown Table 4. The same results were observed in the assessment of single- and multi-unit MSNA incidents. As shown in Table 4, the firing probability of single-unit MSNA in the SS and VSS group was significantly augmented compared with the S group. The multiple spike incidence in the VSS group was significantly augmented compared with the other groups. Figure 3 shows the distribution of cardiac intervals in which there were one, two, three, or four single-unit spikes firing. In the VSS group, there was a significant decrease in the percentage of cardiac intervals that contained one spike compared with the other groups. The distribution of cardiac intervals in which two and three spikes occurred tended to increase in the VSS group. Moreover, significant augmentation was observed in the proportion of cardiac intervals that had four spikes in the VSS group compared with other groups. However, the distribution of cardiac intervals according to spike firings was similar between the S and SS groups.

**Table 4:** **Comparison of sympathetic nerve activity according to OSAS severity**.

	**S**	**SS**	**VSS**
**MULTI-UNIT MSNA**			
BF (bursts/min)	50.6 ± 9.71	56.3 ± 12.6^*^	64.6 ± 12.0^*^^#^
BI (bursts/100 heart beats)	77.4 ± 12.9	82.6 ± 13.2	89.2 ± 8.57^*^^#^
**SINGLE-UNIT MSNA**			
SF (spikes/min)	51.5 ± 10.8	59.0 ± 8.85^*^	73.2 ± 10.9^*^^#^
SI (spikes/100 heart beats)	78.4 ± 15.6	86.5 ± 13.6^*^	101± 15.7^*^^#^
Firing probability (%)	50.4 ± 10.8	55.4 ± 9.67^*^	60.4 ± 10.1^*^
Multiple spike incidence (%)	37.5 ± 10.1	40.9 ± 10.2^*^	49.2 ± 11.2^*^^#^

*Values are means ± SD. S, SAS group; SS, severe SAS group; VSS, very severe SAS group; BF, burst frequency (bursts/min); BI, burst incidence (bursts/100 heart beats); SF, spike firing frequency (spikes/min); SI, spike firing incidence (spikes/100 heart beats)*.

* *p < 0.05, compared to S group*;

# *p < 0.05, compared to SS group*.

**Figure 3:**
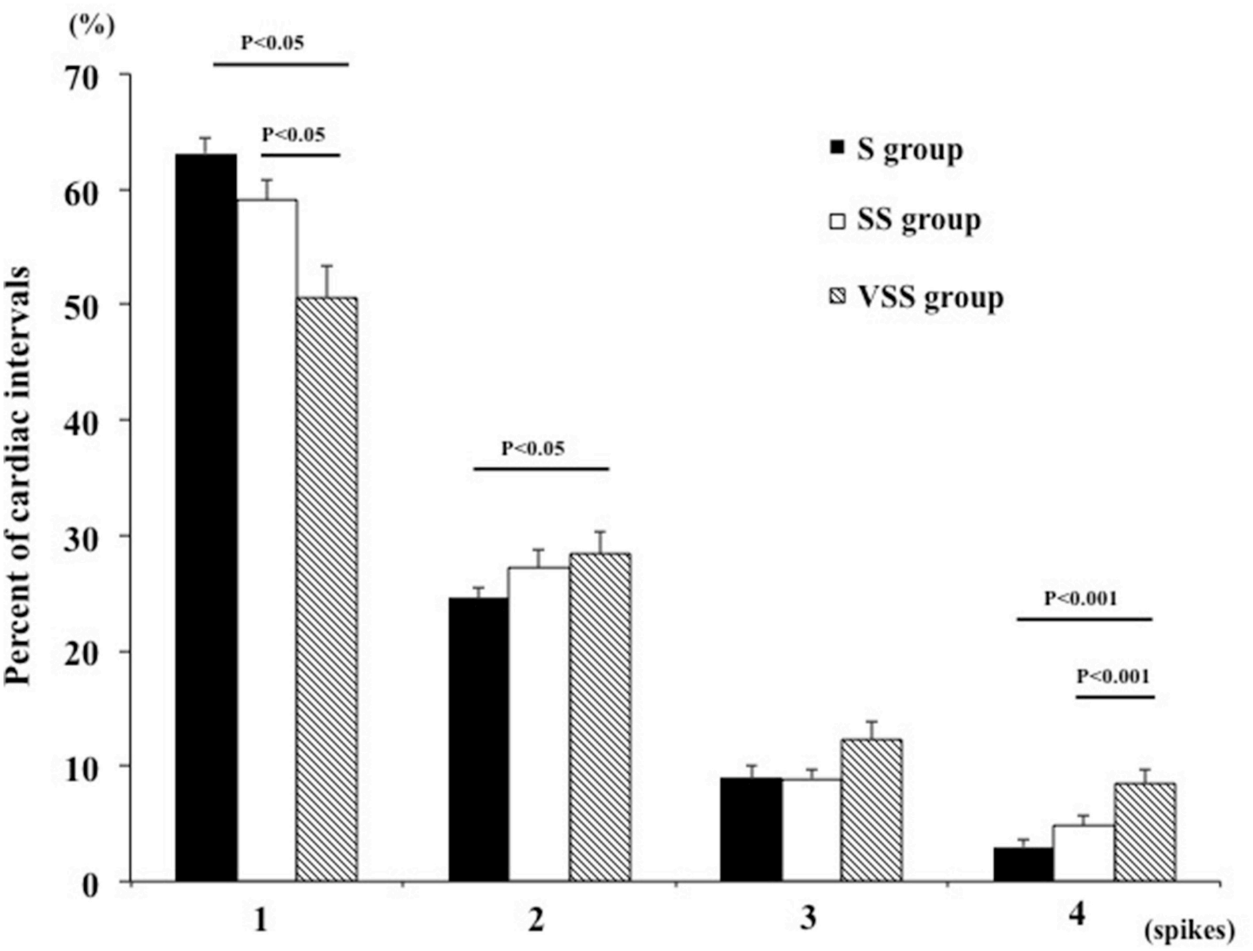
**Percentages of cardiac intervals in which one, two, three, or four single-unit spikes were calculated separately**. In the VSS group, there was a significant decrease in the percentage of cardiac intervals that contained one spike compared with the other groups, and a significant increase was observed in the proportion of cardiac intervals that had four spikes compared with the other groups. There were no statistical differences in the firing properties of the S and SS groups. S group, AHI of 5–30 events/h; SS group, AHI of 30–55 events/h; VSS group, AHI of >55 events/h. AHI, apnea-hypopnea index.

## Discussion

This is the first report to examine the relationship between AHI and single- and multi-unit MSNA in OSAS patients. The novel and important findings from this study are that (1) BMI, blood pressure, and single- and multi-unit MSNA were significantly associated with AHI, but single-unit MSNA was the most significantly correlated with AHI as determine by multivariate regression analysis and (2) single- and multi-unit MSNA increased according to AHI severity. However, patients with an AHI of >55 events/h had a higher proportion of multiple firing spikes than did the other groups, although the results from patients with an AHI of 30–55 events/h were not significantly different than the patients with an AHI of <30 events/h. These results suggest that in intense sympathoexcitation states like severe OSAS, single-unit MSNA is useful to evaluate sympathetic outflow to the periphery.

### Classification of OSAS severity and sympathetic nerve activity

Generally, sleep apnea is classified as no sleep apnea (AHI 5–15), mild OSA (AHI 15–30), moderate OSA, and severe OSA (AHI >30; The Report of an American Academy of Sleep Medicine Task Force, 1999). Previous studies have demonstrated that OSAS mortality is increased in patients with an AHI of >30 events/h, and this can partly be attributed to augmented sympathetic activity (Chesson et al., 2003; Gami et al., 2005). In this study, multi-unit MSNA increased with AHI severity. Multi-unit MSNA is a reliable method with which to assess sympathetic nerve activity in humans, and augmented multi-unit MSNA has been shown to correlate with mortality in HF patients (Barretto et al., 2009). High sympathetic nerve activity is a leading cause of cardiovascular diseases and can contribute to higher mortality in patients with HF as well as chronic kidney disease (Penne et al., 2009). In addition, we found that the probability of multiple firings in single-unit MSNA was significantly increased in patients with an AHI score > 30 events/h (Table 4). Lambert et al. (2011) observed that the incidence of multiple firing in single-unit MSNA was associated with cardiac noradrenaline spillover in humans. These results suggest that high mortality in OSAS patients with an AHI of >30 events/h may be attributed to the increased probability of multiple spikes firing during one cardiac interval.

The results also showed that the distribution of each multiple spike was significantly augmented in patients with high AHIs (>55 events/h) compared with other OSAS patients. There were two reasons to determine the AHI cutoff of 55 events/h. One was that the mean AHI + standard deviation in this study was 55.9 events/h. Another was that the percentage of four spikes firing within one cardiac interval in patients with an AHI of >55 events/h was significantly higher than that in patients with an AHI of <55 events/h (*p* = 0.02). However, patients with an AHI of >54 events/h were not significantly different from those with an AHI of <54 events/h (*p* = 0.07). These results suggest that patients with high AHIs (>55 events/h) have a high sympathoexcitation state, which may contribute to higher mortality.

### Assessment of single-unit MSNA in OSAS patients

Previous studies, including our own, have reported that single-unit MSNA is a more accurate and reliable assessment of sympathetic nerve activity in both healthy and diseased patients (Macefield et al., 1999; Hogarth et al., 2011; Murai et al., 2012; Hering et al., 2013; Millar et al., 2015). Multi-unit MSNA is limited in that it cannot exceed heart rate, and because it is mostly regulated by arterial baroreceptors, burst occurrence is synchronized with cardiac interval. Single-unit MSNA can assess the multiple firings of individual muscle vasoconstrictor neurons during one cardiac interval, and it is not restricted by heart rate. The most important advantage of single-unit MSNA analysis is the ability to demonstrate multiple spikes firing within one cardiac interval. Single-unit MSNA reveals the sympathetic firing mechanism to the periphery. The first mechanism is an increase in the firing frequency of vasoconstrictor fibers that are already active. The second mechanism is an increase in multiple firing within one cardiac interval. The last mechanism is the recruitment of previously silent fibers. Increased multiple firing incidents within one cardiac interval is a state of intense sympathoexcitation in both healthy subjects (in the Valsalva maneuver) and HF patients (Murai et al., 2012). Elam et al. (2002) reported that in eight OSAS patients, the single-unit MSNA frequency exceeded multi-unit MSNA during one cardiac interval. These findings are consistent with our results in that the number of multiple firing spikes within one cardiac interval increased in very severe OSAS patients. In addition to previous findings, we demonstrated that increased firing characteristics of single-unit MSNA were correlated with AHI severity. Interestingly, our data showed that the distribution of each spike firing did not differ between patients with an AHI of <30 events/h and those with an AHI of 30–55 events/h. However, the single-unit MSNA frequency, incidence, and multiple firing incidence were significantly higher in patients with an AHI of 30–55 events/h than in patients with an AHI of <30 events/h. These findings suggest that the mechanism of the increase in multiple single-unit sympathetic firing within one cardiac interval may be maintained until the patient reaches a state of very severe OSAS. In fact, our data showed that the patients with an AHI of >55 events/h had significantly higher blood pressure than the less severe groups.

### Clinical implications

The augmentation of multi-unit MSNA in OSAS patients, both during sleep and waking hours, is well established. The relationship between OSAS severity and sympathoexcitation is poorly understood, and the underlying mechanism responsible for sympathetic nerve activity in OSAS remains unclear. However, our results strongly suggest that long-term, apnea-induced hypoxia can contribute to increased sympathetic nerve activity. Several studies have demonstrated that continuous positive airway pressure (CPAP) treatment improves left ventricular (LV) function in OSAS patients (Kaneko et al., 2003) and inhibits sympathetic nerve activity (measured by MSNA or overnight urinary norepinephrine excretion). CPAP treatment has also been reported to lower norepinephrine levels (Bradley et al., 2005) and reduce risk after heart transplantation (Arzt et al., 2007); however, not all studies have shown improved LV function (Somers et al., 2008). The results from this study suggest that the reduction in AHI by CPAP treatment is not necessarily accompanied by the inhibition of single-unit MSNA firing. Greenwood et al. (2001) demonstrated that single-unit MSNA frequency is associated with LV hypertrophy. In some OSAS patients, MSNA may not be inhibited by CPAP treatment even though the AHI is reduced; in these cases, multi-unit MSNA underestimates sympathetic firing in very severe OSAS patients. Further studies involving more patients are needed to confirm the effects of CPAP on the single-unit MSNA response to LV function and mortality.

### Limitations

This study has some limitations. First, the sampling size in this study was relatively large, but confounding factors still need to be considered. However, this study had an extremely large sampling size compared with other studies. Second, most of the patients (>50%) had been previously treated with antihypertensive drugs such as angiotensin converting enzymes, angiotensin receptor blockers, calcium channel blockers, or thiazide diuretics. These drugs may affect sympathetic nerve activity, and in fact, some antihypertensive drugs have been reported to inhibit sympathetic nerve activity. However, even in patients treated with these drugs, sympathetic nerve activity was still associated with sleep apnea severity, although the proportion of the patients treated with antihypertensive drugs and/or statins were similar among the three groups. In addition, there have been no reports about these effects on MSNA in OSAS patients, except for CPAP. Our results showed that single-unit MSNA was still higher in severe OSAS patients treated with antihypertensive drugs. Lastly, this study was a cross-sectional study that did not include any morbidity or mortality endpoints. Further studies are needed to elucidate the interaction between single-unit MSNA and cardiovascular mortality in OSAS patients.

## Conclusion

In this study, we found that single- and multi-unit MSNA was significantly associated with OSAS severity. Moreover, single-unit MSNA was the most significantly correlated with AHI. The multiple firing spikes ratio measure by single-unit MSNA was significantly higher in very severe OSAS patients. These results suggest that single-unit MSNA is a more accurate reflection of sleep apnea severity and firing property alterations, especially in patients with very severe OSAS.

## Author contributions

TH, HM: Substantial contribution to the conception or design of the work. HT, YS, YN, YO, TK, SU, TH: Acquisition, analysis, or interpretation of data for work. HM, SK, HF: Revising the work critically for important intellectual content. MT: Final approval of the revision to be published. SK, ST, HM: Agreement to be accountable for all aspects of the work in ensuring that questions related to the accuracy or integrity of any part of the work are appropriately investigated and resolved.

### Conflict of interest statement

The authors declare that the research was conducted in the absence of any commercial or financial relationships that could be construed as a potential conflict of interest.

## Abbreviations

AHI: apnea-hypopnea index
AUC: area under the curve
BF: burst frequency
BI: burst incidence
BMI: body mass index
ESS: Epworth sleepiness scale
MSNA: muscle sympathetic nerve activity
PSG: polysomnography
ROC: receiver operating characteristics curve
SES: sleep efficiency score
SF: spike frequency
S group: SAS group
SI: spike incidence
SREM: stage rapid eye movement
SS group: severe SAS group
VSS group: very severe SAS group.
